# Supplementary material for: DNA assembly for nanopore data storage readout
Source: Nat Commun. 2019 Jul 3;10:2933. doi: 10.1038/s41467-019-10978-4 (PMC6610119; doi:10.1038/s41467-019-10978-4)
Supplement: Supplementary file 1 — Supplementary Information [file 41467_2019_10978_MOESM1_ESM.pdf]

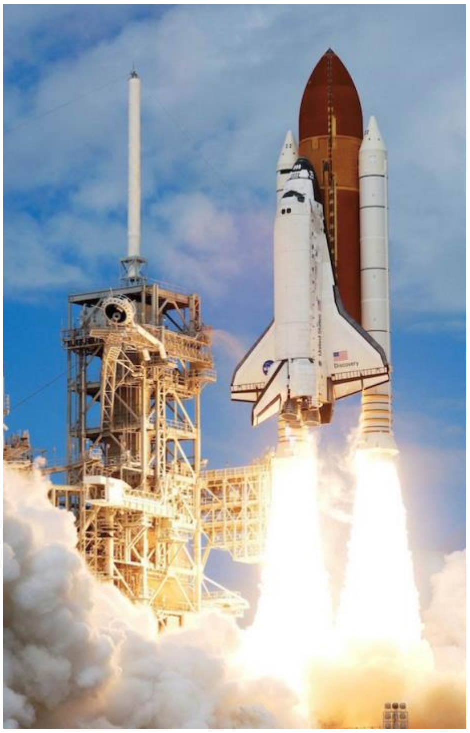

Shuttle.jpg - 113 kB

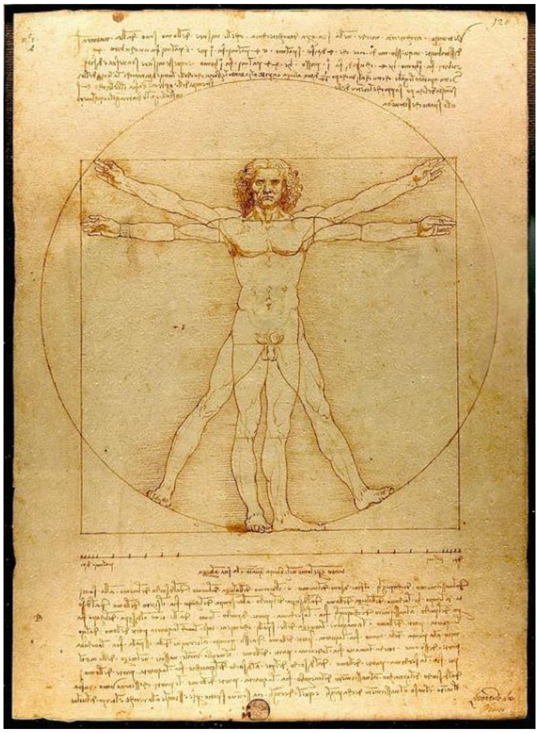

Vitruvian.jpg - 132 kB

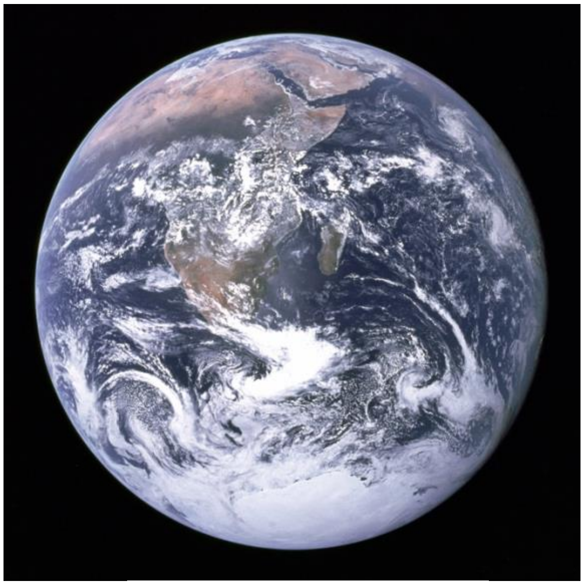

Apollo.jpg - 1,520 kB

Supplementary figure 1 | Three pictures in JPEG format encoded in DNA and sequenced using ONT Nanopore sequencing.

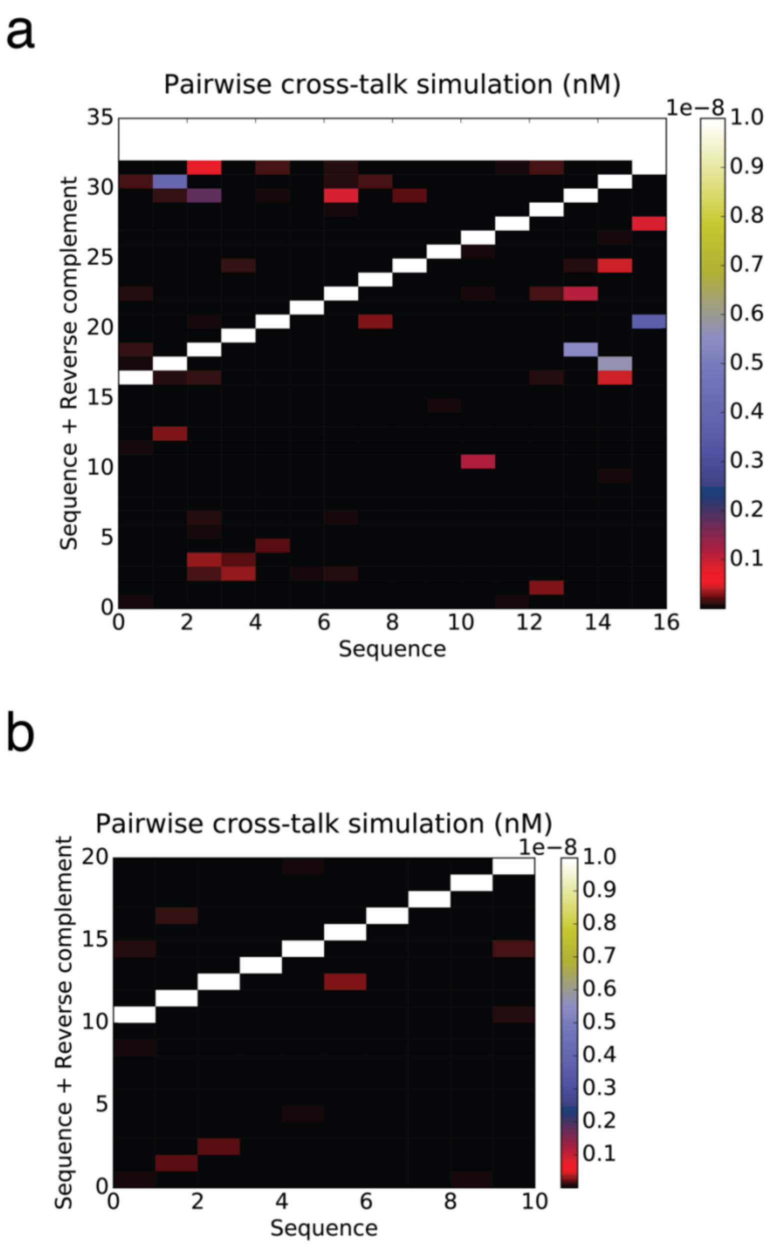

Supplementary figure 2 | Cross-talk simulation for overhang sequences. a, We used a nucleic acid thermodynamic simulation software package (NUPACK) to estimate bound equilibrium concentrations for every pair of overhang sequences with a starting concentration of 10nM at 25°C. The color scale corresponds to these concentrations in molar units. The diagonal line of white data points corresponds to binding between each sequence and its reverse complement. Any colored points outside this diagonal corresponds to undesired cross-talk between overhang sequences. **b,** We implemented an algorithm for sequentially removing overhang sequences with unintended binding. Threshold for unintended interaction was varied based on the number of final fragments necessary for assembly (threshold was set to 1nM for this iteration).

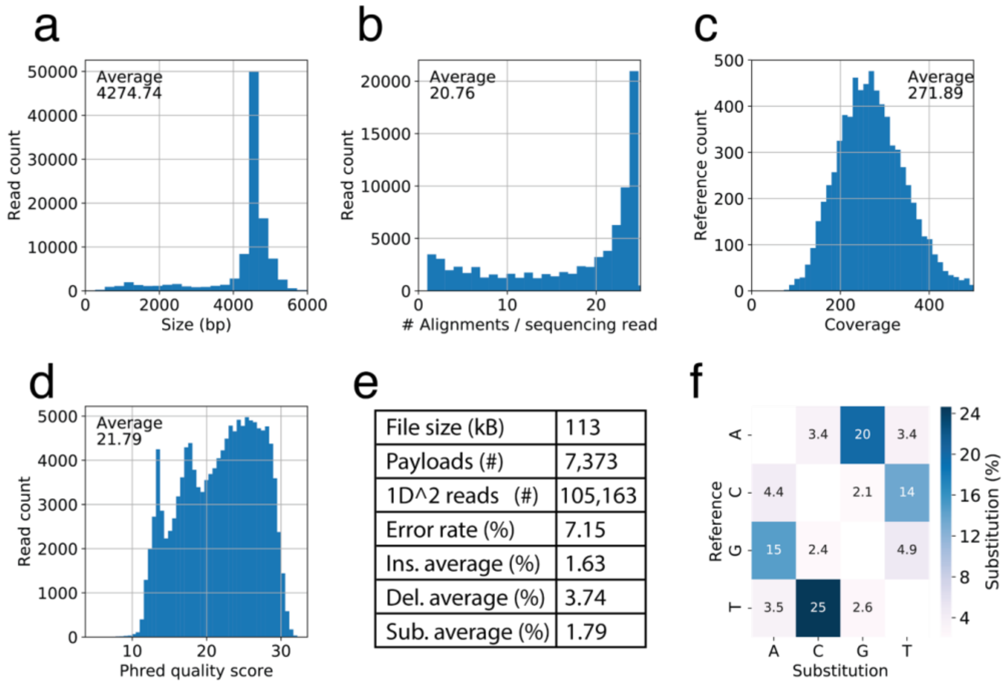

File size (kB)	113
Payloads (#)	7,373
1D^2 reads (#)	105,163
Error rate (%)	7.15
Ins. average (%)	1.63
Del. average (%)	3.74
Sub. average (%)	1.79

Supplementary figure 3 | Sequencing analysis for 113 kB Shuttle file. One MinION flowcells generated 105,163 $1D^2$ reads of the 24-fragment Gibson assembly **a,** Base pair size of sequencing reads matches closely with the assembly size of 4,590 bp. **b,** We aligned each reference payload sequence to the sequencing reads. Each sequencing read resulting in an average of 20.76 alignments to different payloads. Ideally, each read should have 24 alignments. **c,** We found an average sequencing coverage of 271x per payload. **d,** We estimated raw sequencing quality by analyzing the average Phred quality score in each read. **e,** Based on the reads that aligned to payloads, we calculated the average percent error for each base for insertions, deletion and substitutions **(f)** Substitution comparison across different bases revealed strong bias in between purines and pyrimidines.

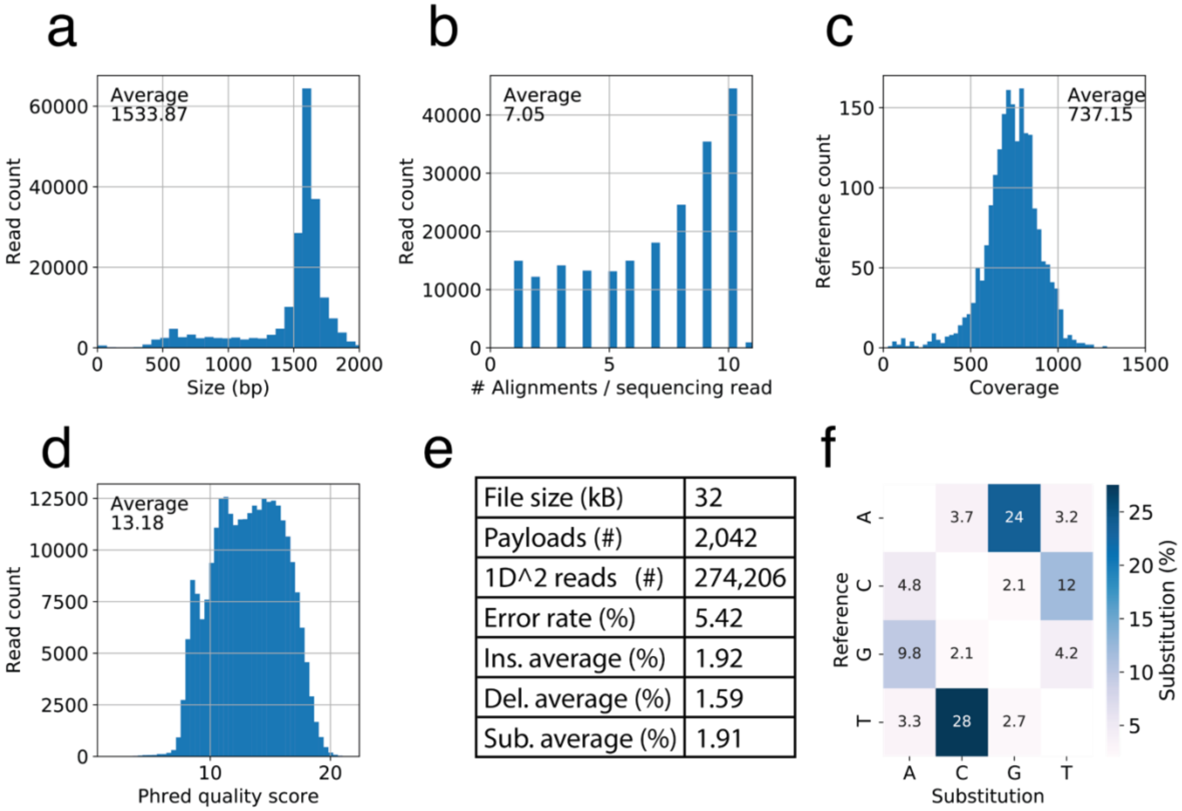

File size (kB)	32
Payloads (#)	2,042
1D^2 reads (#)	274,206
Error rate (%)	5.42
Ins. average (%)	1.92
Del. average (%)	1.59
Sub. average (%)	1.91

Supplementary figure 4 | Sequencing analysis for 32 kB Dishes file. One MinION flowcells generated 274,206 1D^2 reads of the 10-fragment OE-PCR assembly **a,** Base pair size of sequencing reads matches closely with the assembly size of 1,500 bp. **b,** We aligned each reference payload sequence to the sequencing reads. Each sequencing read resulting in an average of 7.05 alignments to different payloads. Ideally, each read should have 10 alignments. **c,** We found an average sequencing coverage of 737x per payload. **d,** We estimated raw sequencing quality by analyzing the average Phred quality score in each read. **e,** Based on the reads that aligned to payloads, we calculated the average percent error for each base for insertions, deletion and substitutions **(f)** Substitution comparison across different bases revealed strong bias in between purines and pyrimidines.

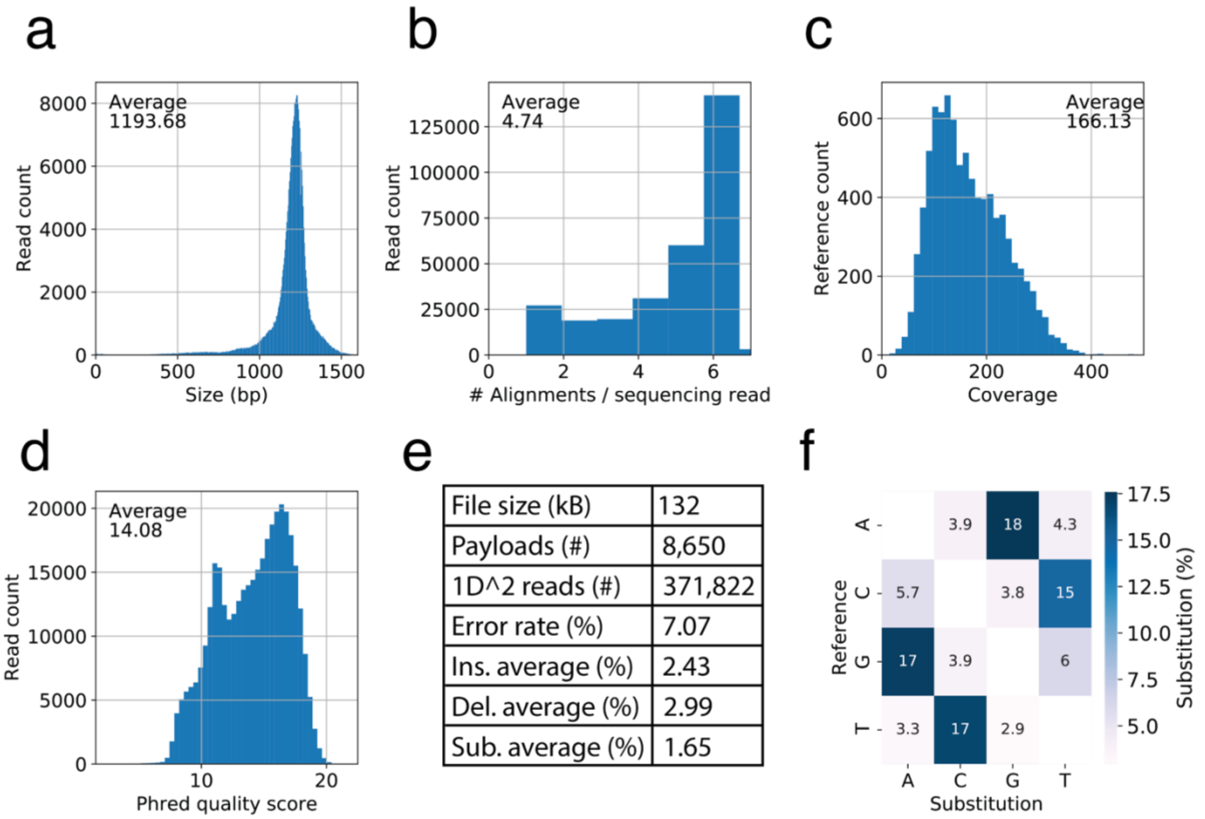

File size (kB)	132
Payloads (#)	8,650
1D^2 reads (#)	371,822
Error rate (%)	7.07
Ins. average (%)	2.43
Del. average (%)	2.99
Sub. average (%)	1.65

Supplementary figure 5 | Sequencing analysis for 132 kB Vitruvian file. One MinION flowcells generated 371,822 1D^2 reads of the 6-fragment Gibson assembly **a,** Base pair size of sequencing reads matches closely with the assembly size of 1,110 bp. **b,** We aligned each reference payload sequence to the sequencing reads. Each sequencing read resulting in an average of 4.74 alignments to different payloads. Ideally, each read should have 6 alignments. **c,** We found an average sequencing coverage of 166x per payload. **d,** We estimated raw sequencing quality by analyzing the average Phred quality score in each read. **e,** Based on the reads that aligned to payloads, we calculated the average percent error for each base for insertions, deletion and substitutions **(f)** Substitution comparison across different bases revealed strong bias in between purines and pyrimidines.

Name	Sequence	Description
AD1	ACATTCCGTGCCATTGGATT	Forward file address
AD2	TCGGCAAATCGTTCCACAAA	Reverse file address
0807_A_FWD_1	CAGGACACTATAACTCCGAAAAGAGACAGTACATTCCGTGCCATTGGATT	Forward primer #1 for adding first assembly overhangs
0807_A_FWD_2	CACAGCCTCGGTAACAGCGCTAGTTTAATTACATTCCGTGCCATTGGATT	Forward primer #2 for adding first assembly overhangs
0807_A_FWD_3	TCCACATCTTTCGGCAGGAGACCACATAAAACATTCCGTGCCATTGGATT	Forward primer #3 for adding first assembly overhangs
0807_A_FWD_4	CCTCTTAATGTGAGCTGCGACCATAGGAGAACATTCCGTGCCATTGGATT	Forward primer #4 for adding first assembly overhangs
0807_A_FWD_5	TACCCAGTCACACCAGAACATGTCGAAAATACATTCCGTGCCATTGGATT	Forward primer #5 for adding first assembly overhangs
0807_A_FWD_6	GAGATCGGATTCTATCGTACGTCTCTCATTACATTCCGTGCCATTGGATT	Forward primer #6 for adding first assembly overhangs
0807_A_REV_1	aattaaactagcgctgttaccgaggctgtgTTTGTGGAACGATTTGCCGA	Reverse primer #1 for adding first assembly overhangs
0807_A_REV_2	tttatgtggtctcctgccgaaagatgtggaTTTGTGGAACGATTTGCCGA	Reverse primer #2 for adding first assembly overhangs
0807_A_REV_3	tctcctatggtcgcagctcacattaagaggTTTGTGGAACGATTTGCCGA	Reverse primer #3 for adding first assembly overhangs
0807_A_REV_4	attttcgacatgttctggtgtgactgggtaTTTGTGGAACGATTTGCCGA	Reverse primer #4 for adding first assembly overhangs
0807_A_REV_5	aatgagagacgtacgatagaatccgatctcTTTGTGGAACGATTTGCCGA	Reverse primer #5 for adding first assembly overhangs
0807_A_REV_6	tgtaggctcatattgtgctcattagtcctgTTTGTGGAACGATTTGCCGA	Reverse primer #6 for adding first assembly overhangs
0807_A_FWD*	CAGGACACTATAACTCCGAA	Forward primer for first assembly amplification.
0807_A_REV*	tgtaggctcatattgtgctc	Reverse primer for first assembly amplification.
0807_B_FWD_1	GATCAAAATGCGACCAGTAAATCAGACGGCCAGGACACTATAACTCCGAA	Forward primer #1 for adding second assembly overhangs
0807_B_FWD_2	TTCAATGAAAGTATAGCCGCCAGTCGATGTCAGGACACTATAACTCCGAA	Forward primer #2 for adding second assembly overhangs
0807_B_FWD_3	GTTCGGGTACTCAAGGATTAATCGCGAGGACAGGACACTATAACTCCGAA	Forward primer #3 for adding second assembly overhangs
0807_B_FWD_4	CATTTACAAAGGACCCGAGATTCACAGATGCAGGACACTATAACTCCGAA	Forward primer #4 for adding second assembly overhangs
0807_B_REV_1	acatcgactggcggctatactttcattgaatgtaggctcatattgtgctc	Reverse primer #1 for adding second assembly overhangs
0807_B_REV_2	tcctcgcgattaatccttgagtacccgaactgtaggctcatattgtgctc	Reverse primer #2 for adding second assembly overhangs
0807_B_REV_3	catctgtgaatctcgggtcctttgtaaatgtgtaggctcatattgtgctc	Reverse primer #3 for adding second assembly overhangs
0807_B_REV_4	gccaacctataccaatccttatgaactcgttgtaggctcatattgtgctc	Reverse primer #4 for adding second assembly overhangs
0807_B_FWD*	GATCAAAATGCGACCAGTAAATCAG	Forward primer for second assembly amplification.
0807_B_REV*	gccaacctataccaatccttatgaa	Reverse primer for second assembly amplification.

Supplementary table 1 | Primer sequences for amplification and assembly of the Space Shuttle file. AD1 & AD2 correspond to the address sequences for the file. There are six primer pairs for the first assembly ‘A’ that are necessary to insert the overhangs for a 6-fragment assembly and an additional pair to amplify the assembly product (0807_A_FWD* & 0807_A_REV*). The first assembly product is then amplified with four primer pairs ‘B; to insert the overhangs for a second 4-fragment assembly and an additional pair to amplify the corresponding assembly product (0807_B_FWD* & 0807_B_REV*).

Name	Sequence	Description
FP1	TGAAACACCTCTAGCACCAG	Forward primer for Group1
RP1	AATCATAGAATTTCGCGGCCTGCTCGACTATGCAAGCGTC	Reverse primer for Group1
FP2	GACGCTTGCATAGTCGAGCAGGCCGCGAAATTCTATGATT	Forward primer for Group2
RP2	ACACACTGCGTCGGACTTCGTATCAAGCGCGGCTCCTTAA	Reverse primer for Group2
FP3	TTAAGGAGCCGCGCTTGATACGAAGTCCGACGCAGTGTGT	Forward primer for Group3
RP3	GCTAGTTCTGCGATCAGTCTCCACGGTTTGTCACGGTCAC	Reverse primer for Group3
FP4	GTGACCGTGACAAACCGTGGAGACTGATCGCAGAACTAGC	Forward primer for Group4
RP4	ATTGACGGAACCTGGCTGTTGTCAACGAATCATGTCGCAT	Reverse primer for Group4
FP5	ATGCGACATGATTCGTTGACAACAGCCAGGTTCCGTCAAT	Forward primer for Group5
RP5	GAATCAAGGCACTCGCGTATCTCATCGCCGTCGGAATAGC	Reverse primer for Group5
FP6	GCTATTCCGACGGCGATGAGATACGCGAGTGCCTTGATTC	Forward primer for Group6
RP6	AGGTTAATTCCGCGTGAGATTGCCACTCAACCAGACGCCA	Reverse primer for Group6
FP7	TGGCGTCTGGTTGAGTGGCAATCTCACGCGGAATTAACCT	Forward primer for Group7
RP7	AATACTGCGTGAGGTCCTGTGTCTAAGGTAGTCCATGCCT	Reverse primer for Group7
FP8	AGGCATGGACTACCTTAGACACAGGACCTCACGCAGTATT	Forward primer for Group8
RP8	AAAGCCTTGTGACCGCTTAATTTCATGCACACCGATCTAC	Reverse primer for Group8
FP9	GTAGATCGGTGTGCATGAAATTAAGCGGTCACAAGGCTTT	Forward primer for Group9
RP9	AAGAGTATCCGGTCACCTGATGCTGTATCAGCTCGACATG	Reverse primer for Group9
FP10	CATGTCGAGCTGATACAGCATCAGGTGACCGGATACTCTT	Forward primer for Group10
RP10	GGACGGATTGACAGTCGGAT	Reverse primer for Group10

Supplementary table 2 | Primer sequences for amplification and assembly of the 365-dishes file (OE-PCR)..
